# Supplementary figures and images for: Identifying risk zones and landscape features that affect common leopard depredation
Source: PeerJ. 2024 May 31;12:e17497. doi: 10.7717/peerj.17497 (PMC11146323; doi:10.7717/peerj.17497)

### Correlation Matrix of Variables

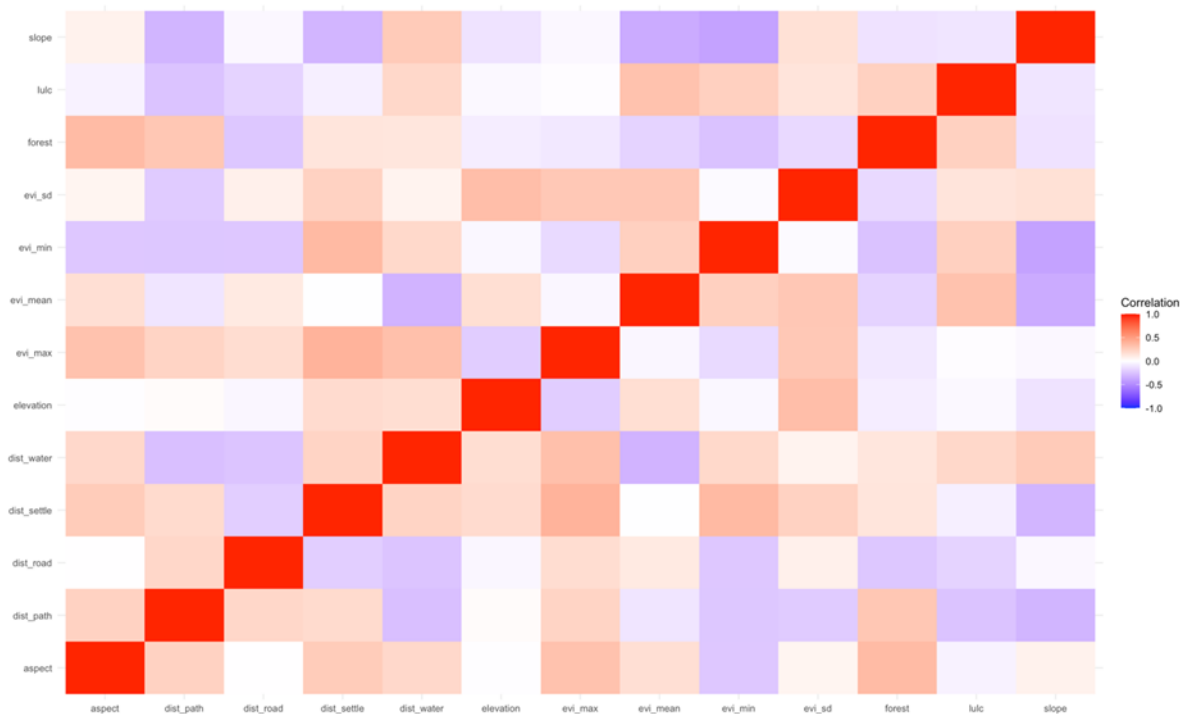

Supplement: Supplemental Information 1 [file peerj-12-17497-s001.pdf]
